# Reversal of Fragile X Phenotypes by Manipulation of AβPP/Aβ Levels in *Fmr1^KO^* Mice

**DOI:** 10.1371/journal.pone.0026549

**Published:** 2011-10-26

**Authors:** Cara J. Westmark, Pamela R. Westmark, Kenneth J. O'Riordan, Brian C. Ray, Crystal M. Hervey, M. Shahriar Salamat, Sara H. Abozeid, Kelsey M. Stein, Levi A. Stodola, Michael Tranfaglia, Corinna Burger, Elizabeth M. Berry-Kravis, James S. Malter

**Affiliations:** 1 Waisman Center for Developmental Disabilities, University of Wisconsin, Madison, Wisconsin, United States of America; 2 Department of Neurology, University of Wisconsin, Madison, Wisconsin, United States of America; 3 Department of Pathology & Laboratory Medicine, University of Wisconsin, Madison, Wisconsin, United States of America; 4 Department of Pediatrics, Rush University Medical Center, Chicago, Illinois, United States of America; 5 Department of Biochemistry, Rush University Medical Center, Chicago, Illinois, United States of America; 6 Department of Neurological Sciences, Rush University Medical Center, Chicago, Illinois, United States of America; 7 FRAXA Research Foundation, Newburyport, Massachusetts, United States of America; Federal University of Rio de Janeiro, Brazil

## Abstract

Fragile X syndrome (FXS) is the most common form of inherited intellectual disability and the leading known genetic cause of autism. Fragile X mental retardation protein (FMRP), which is absent or expressed at substantially reduced levels in FXS, binds to and controls the postsynaptic translation of amyloid β-protein precursor (AβPP) mRNA. Cleavage of AβPP can produce β-amyloid (Aβ), a 39–43 amino acid peptide mis-expressed in Alzheimer's disease (AD) and Down syndrome (DS). Aβ is over-expressed in the brain of *Fmr1^KO^* mice, suggesting a pathogenic role in FXS. To determine if genetic reduction of AβPP/Aβ rescues characteristic FXS phenotypes, we assessed audiogenic seizures (AGS), anxiety, the ratio of mature versus immature dendritic spines and metabotropic glutamate receptor (mGluR)-mediated long-term depression (LTD) in *Fmr1^KO^* mice after removal of one *App* allele. All of these phenotypes were partially or completely reverted to normal. Plasma Aβ_1–42_ was significantly reduced in full-mutation FXS males compared to age-matched controls while cortical and hippocampal levels were somewhat increased, suggesting that Aβ is sequestered in the brain. Evolving therapies directed at reducing Aβ in AD may be applicable to FXS and Aβ may serve as a plasma-based biomarker to facilitate disease diagnosis or assess therapeutic efficacy.

## Introduction

FXS is an X chromosome-linked disorder characterized by highly variable intellectual disability, autistic-like behavior and seizures [1]. In the vast majority of cases, FXS results from a >200 trinucleotide (CGG) repeat expansion in the 5′-UTR of the *FMR1* gene [2] leading to transcriptional silencing and loss or reduction of expression of FMRP [3]. FMRP is a multi-functional mRNA binding protein involved in the dendritic transport, localization and translational regulation of several hundred mRNA ligands [4]–[9]. In the absence of FMRP, dendritic spine morphology and function are abnormal [10]. Thus, FXS is likely caused by the inappropriate, post-synaptic expression of one or more FMRP mRNA targets. We have previously demonstrated that post-synaptic translation of *App* mRNA is regulated by FMRP through a mGluR_5_-dependent pathway. In the absence of FMRP, excess AβPP and its catabolites Aβ_1–40_ and Aβ_1–42_, accumulate in the brains of middle-aged *Fmr1^KO^* mice [11].

There is very limited data regarding the roles of AβPP mRNA, protein and catabolites in persons with FXS. One group showed elevated *App* mRNA in the cerebral cortex, hippocampus and cerebellar cortex in *Fmr1^KO^* mice [12], but we have not observed differences in cortical synaptoneurosomes [11]. Increased Aβ levels would predict an increased incidence of AD pathology in aged FXS individuals, which has not been observed in neuropathological analyses of a very small number of specimens [13]–[15]. Likewise, there are no reports of an increased incidence of AD in FXS, but it is difficult to assess age-related dementia in the mentally retarded and very few elderly individuals with FXS have been studied in significant numbers. Recent data suggests that AβPP or its proteolytic derivatives may be aberrantly expressed in children with severe autism [16], [17], which is extremely prevalent in FXS (67% of males and 23% of females) [18] and DS (7%) [19]. Thus, the increased production or altered processing of AβPP may contribute to the intellectual disabilities observed in all of these disorders.

Herein, we demonstrate rescue of several *Fmr1^KO^* phenotypes in model mice by genetic modulation of AβPP/Aβ levels. Treatment of primary neurons with Aβ_1–42_ rapidly triggered extracellular-regulated kinase (ERK) signaling and altered the translation of multiple FMRP target mRNAs including *App*. Conversely, anti-Aβ antibody decreased dendritic AβPP levels. These data suggest that Aβ modulates its own production through a positive feedback loop. Finally, we show that plasma Aβ_1–42_ is significantly reduced while cerebral Aβ_1–42_ is likely increased in full-mutation FXS males. These data suggest evolving therapies directed at reducing Aβ in AD may be applicable to FXS and plasma Aβ_1–42_ may be a biomarker for disease severity and drug efficacy in FXS.

## Results

### Genetic Modulation of AβPP Levels in *Fmr1^KO^* Mice Reduces AGS

**Figure 1:**
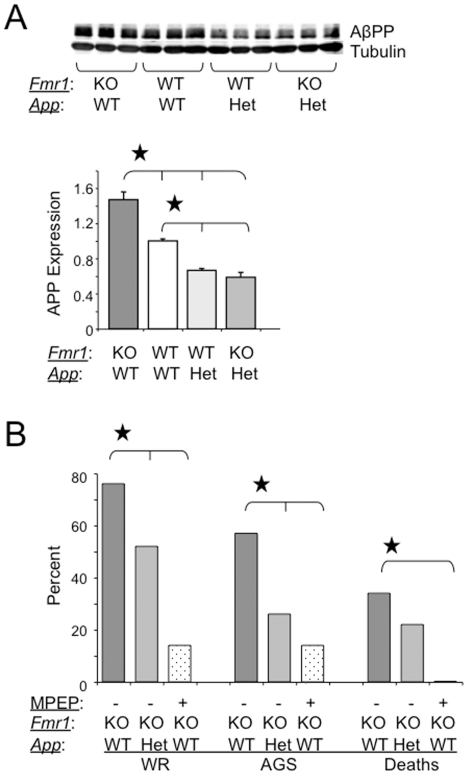
AGS are rescued by genetic manipulation of *App* or mGluR_5_ blockade. (A) western blot analyses of AβPP levels in *Fmr1^KO^*, WT, *App^HE^* ^T^ and *Fmr1^KO^/App^HET^* mice (n = 3 male mice per strain, 1 month old). Statistics: one-way ANOVA p<0.0001, F = 53.95. Stars (★) denote statistically different levels by Student T-test analyses and by Bonferroni's multiple comparison test (p<0.05). Error bars represent SEM. (B) Assessment of WR, AGS and death rates in *Fmr1^KO^* mice (age P21, n = 58)) after genetic manipulation of AβPP levels (n = 23) or treatment with MPEP (n = 14). Stars (★) denote rates that are statistically different from untreated mice (p<0.5) by Fisher exact tests.

Compared to WT, *Fmr1^KO^* mice exhibit AGS [20], altered anxiety [21]–[23] and dendritic spine dysmorphogenesis [24]–[26], features shared with patients with FXS [1], [27], [28]. In order to establish if AβPP or Aβ directly contributed to FXS pathogenesis, we modulated AβPP and Aβ expression in *Fmr1^KO^* mice. Thus, we generated *Fmr1^KO^*/*App^HET^* and *Fmr1^KO^*/*App^KO^* mice all in a pure C57BL/6 background to evaluate the effects of genetic modulation of AβPP and Aβ expression. Western blot analyses confirmed that AβPP levels were reduced by 50% in *Fmr1^KO^*/*App^HET^* mice (Figure 1A). At 21 days of age, animals were evaluated for AGS. Wild running (WR) and seizures decreased 32% and 54%, respectively, in *Fmr1^KO^*/*App^HET^* mice (Figure 1B). These data suggest that seizures are enhanced when AβPP is absent or over-expressed and that AβPP or one of its metabolites significantly contributes to the AGS phenotype seen in *Fmr1^KO^* mice.

Pharmacological antagonists directed at or genetic reduction of mGluR_5_ correct many *Fmr1^KO^* phenotypes [22], [26], [29]–[31]. We compared the efficacy of mGluR_5_ antagonists with genetic manipulation of AβPP/Aβ in reducing AGS in *Fmr1^KO^* mice. A 30 min pretreatment with 2-methyl-6-(phenylethynyl)pyridine hydrochloride (MPEP) delivered by I.P. injection reduced WR, AGS and deaths by 82%, 75% and 100%, respectively (Figure 1B). An alternative mGluR_5_ antagonist, fenobam, reduced WR, AGS and deaths to 0% (data not shown).

### *Fmr1^KO^*/*App^HET^* Mice Lack FXS Behavioral, Dendritic Spine and mGluR-LTD Phenotypes

**Figure 2:**
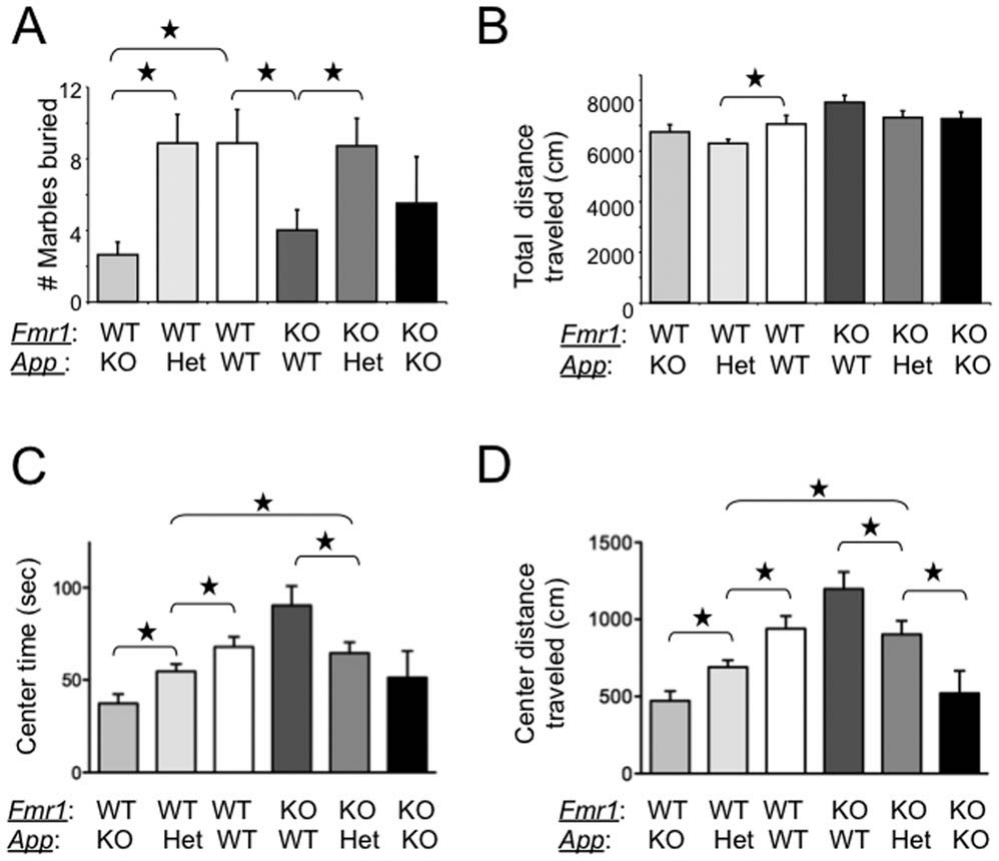
Rescue of behavioral phenotypes in adult *Fmr1^KO^* mice by genetic manipulation of *App*. (A) male *App^KO^* (n = 8), *App^HET^* (n = 14), WT (n = 7), *Fmr-1^KO^* (n = 8), *Fmr1^KO^/App^HET^* (n = 10) and *Fmr1^KO^/App^KO^* (n = 4) mice (8–10 weeks old) were assessed for marble burying activity. Statistics: one-way ANOVA p<0.022, F = 2.95. (B, C and D) Mice were assessed for anxiety levels and locomotion in the open field. Both genders were included as the males and females exhibited equivalent locomotion and anxiety in the open field [*App^KO^* (n = 11), *App^HET^*(n = 23), WT (n = 15), *Fmr1^KO^* (n = 14), *Fmr-1^KO^/App^HET^* (n = 18) and *Fmr1^KO^/App^KO^* (n = 7)]. (B) Total distance (cm) traveled is plotted against mouse strain. Statistics: one-way ANOVA p<0.0005, F = 4.59. (C) Time (sec) spent in the center of the arena is plotted versus mouse strain. Statistics: one-way ANOVA p<0.0001, F = 5.98. (D) Distance (cm) traveled in the center of the arena is plotted versus mouse strain. Statistics: one-way ANOVA p<0.0001, F = 9.17. Stars (★) denote statistically different levels by Student T-test analyses (p<0.5). All error bars represent SEM.

Hyperactivity, social anxiety and autistic-like behaviors are characteristic features of FXS [1]. We first assessed marble burying in these mice as a measure of repetitive behavior [32]. *Fmr1^KO^* male mice buried significantly fewer marbles than WT (p = 0.04) (Figure 2A), which was rescued in male *Fmr1^KO^/App^HET^* mice (statistically different compared to *Fmr1^KO^*, p = 0.03; not different from WT, p = 0.95). Thus, repetitive digging is a normal mouse behavior that can be rescued in *Fmr1^KO^* male mice by genetically reducing AβPP/Aβ levels.

Next, we assessed hyperactivity (Figure 2B) and anxiety (Figure 2C, D) in the open field test. *Fmr1^KO^* exhibit increased center time (reflecting a loss of anxiety) but equivalent locomotion in the open field compared with WT mice [21], [22]. *App^HET^* and *App^KO^* mice exhibited significantly more anxiety/thigmotaxis than WT mice (Figure 2C). The increased thigmotaxis in the *App^HET^* is partially due to decreased locomotion compared with WT mice (Figure 2B); however, total distance traveled was equivalent between *App^KO^* and *App^HET^* indicating that decreased AβPP /Aβ levels correlate with increased anxiety. *Fmr1^KO^/App^HET^* mice exhibited equivalent center time as WT mice and significantly less than *Fmr1^KO^* mice indicating that thigmotaxis was rescued. Total distance traveled in the open field was not statistically different between *Fmr1^KO^/App^HET^*, WT and/or *Fmr1^KO^* mice indicating equivalent locomotion.

**Figure 3:**
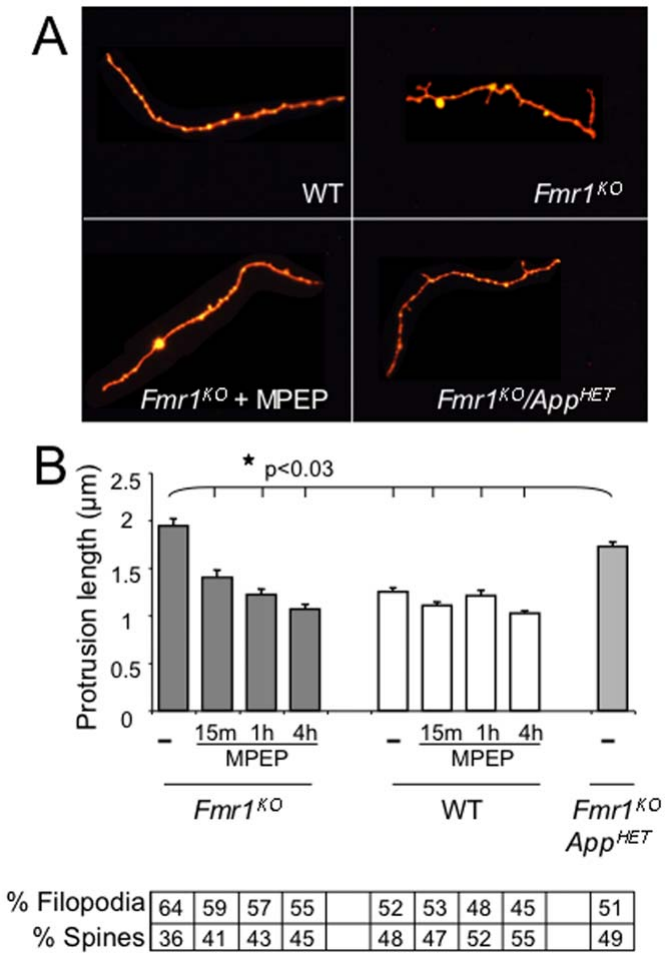
Dendritic spine morphology is partially rescued in *Fmr1^KO^/App^HET^* or MPEP treated *Fmr1^KO^* mice. (A) Representative fluorescent images of primary cultured neurons prepared from WT (upper left), *Fmr1^KO^* (upper right and lower left) and *Fmr1^KO^/App^HET^* (lower right) embryos stained with DiI and visualized by fluorescent microscopy (100× objective). The arrows denote dendritic spines. (B) The lengths of dendritic protrusions were quantitated with StereoInvestigator software and plotted against mouse strain/treatment. The percentage of filopodia versus spines for each condition is given below the histogram. Statistics: one-way ANOVA comparison of the three genotypes (untreated) p<0.0001, F = 27.18. All genotypes are statistically different from each other by Student T-Test and Bonferroni's multiple comparison tests. Two-way ANOVA comparison of WT versus *Fmr1^KO^* ± MPEP: p<0.0001, F = 12.89 (interaction), F = 35.01 (genotype) and F = 27.62 (MPEP). The untreated and 15 min MPEP treated WT spines are statistically different from the corresponding *Fmr1^KO^* spines by the Bonferroni multiple comparison test (p<0.5). Stars (★) denote statistically different spine lengths by Student T-test analyses (p<0.5). Error bars indicate SEM [*Fmr1^KO^*: untreated (n = 746 spines), 15 min MPEP (n = 263), 1 hr MPEP (n = 300), 4 hr MPEP (n = 293); WT: untreated (n = 994), 15 min MPEP (n = 535), 1 hr MPEP (n = 373), 4 hr MPEP (n = 1221); *Fmr1^KO^/App^HET^* (n = 2469)].

Pathological examination of brains from FXS patients has shown an increased density of long and tortuous dendritic spines suggesting a delay in spine maturation [15], [33]. We assessed dendritic spine length in primary cultured neurons and found a statistically significant 1.6-fold increase in protrusion length in the *Fmr1^KO^ cells* compared to WT (Figure 3A, B) in agreement with the literature [24]–[26]. A 4 hr treatment with mGluR_5_ antagonists (MPEP or fenobam) rescued the spine/filopodia ratio in *Fmr1^KO^* mice to WT levels [26]. Likewise, we observed that both dendritic spine length and the percentage of filopodia in primary cultured *Fmr1^KO^* neurons were reverted to WT levels within 15 min of MPEP treatment (Figure 3B). Average protrusion length was reduced by 11% in the *Fmr1^KO^*/*App^HET^* cells (statistically significant p<0.03 compared to *Fmr1^KO^*), and the percentage of filopodia (immature spines) versus mature spines was completely reverted to WT levels. Similarly, minocycline treatment of *Fmr1^KO^* neurons has been reported to rescue the percentage of mature spines to WT levels albeit without a significant change in dendritic protrusion length [24].

**Figure 4:**
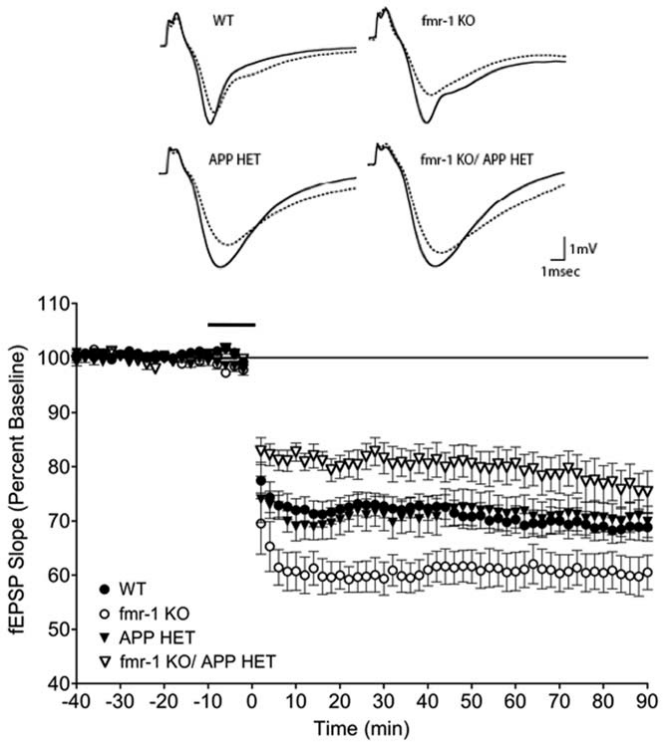
Rescue of mGluR-dependent synaptic depression in the CA1 in *Fmr1^KO^*/*App^HET^* mice. (Top) Representative fEPSP taken before the addition of DHPG (solid lines) and at the end of the recordings (dotted lines). (Bottom) Time course of CA1 fEPSP slope after incubation with DHPG (50 µM, 10 min) in hippocampal slices from WT (black circles) (n = 20), *Fmr1^KO^* (white circles) (n = 13), *App^HET^* (black triangles) (n = 15) and *Fmr1^KO^/App^HET^* (white triangles) (n = 10) male mice (3 months old). WT, *App^HET^* and *Fmr1^KO^/App^HET^* were all statistically different from *Fmr1^KO^* by two-way ANOVA/Bonferroni multiple comparison tests. WT versus *App^HET^*, p = 0.92; WT versus *Fmr1^KO^*, p<0.006; WT versus *Fmr1^KO^/App^HET^*, p<0.02; *App^HET^* versus *Fmr1^KO^/App^HET^*, p<0.04; *App^HET^* versus *Fmr1^KO^*, p = 0.02; *Fmr1^KO^* versus *Fmr1^KO^/App^HET^*, p<0.0002. Error bars indicate SEM.

*Fmr1^KO^* mice exhibit enhanced hippocampal mGluR-long term depression (LTD), which requires rapid protein synthesis [34], [35]. We assessed hippocampal mGluR-LTD by field recordings in 3-month-old male WT, *Fmr1^KO^*, *App^HET^* and *Fmr1^KO^*/*App^HET^* mice. mGluR-dependent synaptic depression was enhanced in the CA1 in *Fmr1^KO^* slices, equivalent in WT and *App^HET^* and reduced in *Fmr1^KO^*/*App^HET^* (statistically significant p<0.0002 comparing *Fmr1^KO^*/*App^HET^* and *Fmr1^KO^*) (Figure 4). The time course of CA1 fEPSP slopes after DHPG treatment reveals a significant difference between *Fmr1^KO^* animals relative to wild type, *App^HET^* and *Fmr1^KO^*/*App^HET^* mice. Input/output relationships and paired-pulse facilitation (ppf) were analyzed to assess the basal synaptic properties of the different groups (Figure S1), and did not show significant changes between experimental groups indicating unaltered synaptic transmission in the *Fmr1^KO^* hippocampus.

### Aβ_1–42_ Alters Dendritic Expression of FMRP Targets

**Figure 5:**
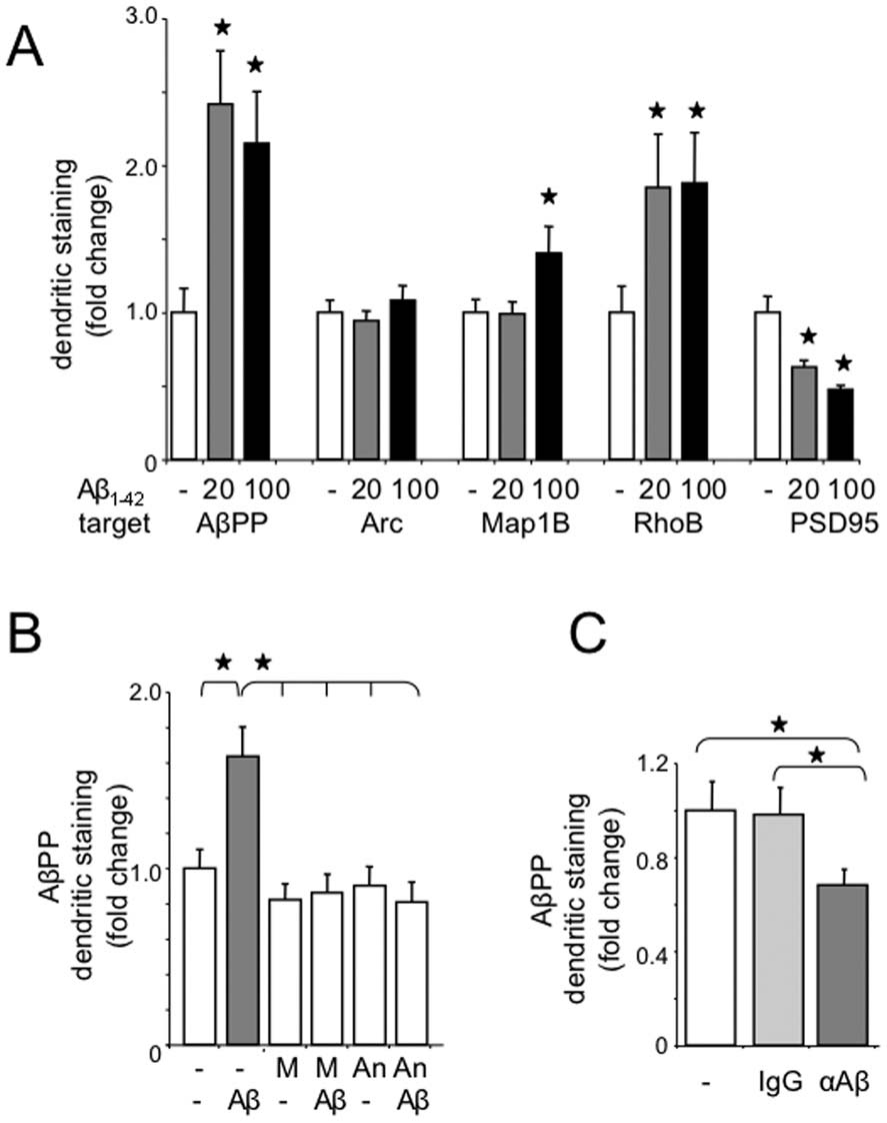
Aβ_1–42_ alters dendritic protein levels. (A) WT neuronal cells treated with vehicle, 20 or 100 nM Aβ_1–42_ for 1 hr followed by fixation and staining for AβPP, Arc/Arg, Map1B, RhoB and PSD95 and analyses by confocal fluorescent microscopy. (B) WT neuronal cells pre-treated with vehicle, 2.5 µM MPEP or 40 µM anisomycin for 15 min prior to treatment with 20 nM Aβ_1–42_ for 1 hr and fixation and staining for AβPP. Statistics: one-way ANOVA p<0.0001, F = 7.04. All treatments are statistically different from the 20 nM Aβ_1–42_ treatment by the Bonferroni multiple comparison test (p<0.05). A minimum of 1793 particles were analyzed per treatment cohort. (C) WT neuronal cells treated with mouse IgG or anti-Aβ antibody in transwells for 3 days prior to fixation and staining for AβPP. Statistics: one-way ANOVA p<0.05, F = 3.03. A minimum of 2,644 particles were analyzed per treatment cohort. Stars (★) denote statistical differences by Student T-test analyses (p<0.5). Error bars indicate SEM.

To begin to understand how excessive production and/or processing of AβPP mediates cell signaling events, we examined dendritic expression of select FMRP targets after treating WT primary neurons with soluble Aβ_1–42_. There was a >2-fold increase in the expression of AβPP, no change in Arc, 40% increase in Map1B, 85% increase in RhoB and 50% decrease in PSD95 (Figure 5A). The Aβ_1–42_-mediated increase in dendritic AβPP could be blocked with MPEP or anisomycin indicating that it was mGluR_5_- and translation-dependent (Figure 5B). Conversely, reduction of Aβ in the cell culture media by treatment with anti-Aβ antibody through a transwell reduced dendritic AβPP expression by 31% (Figure 5C). We utilized transwells to avoid direct contact between the antibody and the cells as direct application of anti-Aβ to the culture media dramatically increased AβPP expression presumably due to cell signaling events initiated by anti-Aβ binding to cell surface receptors (data not shown). MPEP and Aβ_1–42_ altered phosphorylated levels of ERK (Figure S2), suggesting that the mitogen activated protein kinase pathway is as an intermediate in Aβ-mediated AβPP translation.

### Aβ_1–42_ Levels are Abnormal in Humans with FXS

**Figure 6:**
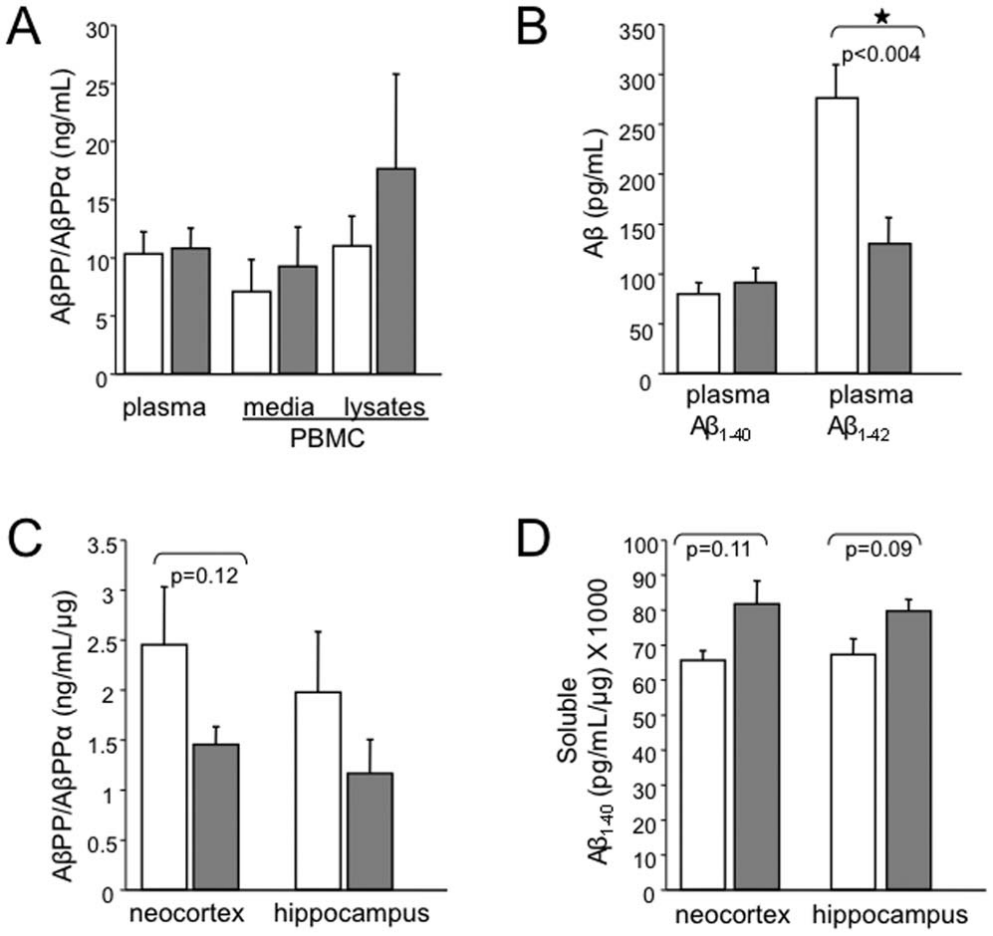
AβPP/Aβ are abnormal in blood and brain from FXS patients. (A) AβPPα levels in control (n = 7) and FXS (n = 10) plasma and PBMC culture media (n = 7 controls and 4 FXS) and AβPP in PBMC (n = 7 controls and 5 FXS). For the plasma and PBMC culture media data, AβPPα is expressed as ng/mL and for the PBMC lysate measurements, AβPP is expressed as ng/mL/µg lysate. (B) Aβ_1–40_ and Aβ_1–42_ levels in control (n = 7) and FXS (n = 10) plasma. For Aβ_1–42_, p<0.004 as determined by Student T-Test analyses. (C) AβPP/AβPPα in control (n = 3) and FXS (n = 4) neocortex and hippocampus. Controls were gender- and age-matched to FXS donors by the University of Maryland Brain Bank. All of the donors were Caucasian males and their ages ranged from 21–85 years old. (D) Soluble Aβ_1–40_ levels in control (n = 3) and FXS (n = 4) neocortex and hippocampus. White bars = control samples and gray bars = FXS. Error bars represent SEM.

We are unaware of any published data evaluating AβPP or its products in the blood of adult FXS patients. We found that plasma AβPP/AβPPα and Aβ_1–40_ levels were comparable in adult FXS patients and controls (Figure 6A, B). These results are distinct from those previously observed in children [16] and suggest that AβPP expression and processing decrease with age. Indeed, AβPPα levels are higher in children 7 years and younger than those 10 years and older [16]. While plasma Aβ_1–40_ levels were unchanged between FXS and controls, Aβ_1–42_ was significantly lower in the FXS group (2.1-fold decrease, p<0.004) (Figure 6B). As seen in other amyloidogenic diseases, the Aβ_1–42_/Aβ_1–40_ ratio (1.4∶1) in blood plasma was substantially reduced compared to controls (3.4∶1) suggesting decreased clearance from the brain. There was no statistically significant increases in cell-associated AβPP, secreted AβPPα nor *APP*_695/751/770_ mRNA in peripheral blood mononuclear cells (PBMC) samples from FXS and control donors (Figure 6A and data not shown).

We next assessed AβPP/AβPPα, Aβ_1–40_ and Aβ_1–42_ in hippocampal and neocortical control and FXS autopsy brain tissue. The data represents analyses of four FXS and three control brains. There is a strong trend toward increased soluble Aβ_1–40_ in lysates of FXS brain samples (Figure 6D) while AβPP/AβPPα levels are reciprocally decreased (Figure 6C) in both the neocortex and hippocampus. Despite the small sample size due to the unavailability of tissue, the 1.7-fold decrease in AβPP/AβPPα in neocortex approaches statistical significance. These data suggest that the brain may act as a sink for Aβ and that lower blood plasma levels may indicate increased brain deposition. These results are similar to those in *Fmr1^KO^* mice, which exhibited elevated Aβ in the brain [11]. Murine Aβ levels in blood plasma were below the ELISA detection limit and could not be determined.

These data strongly support the hypothesis that modest over-expression of AβPP and/or Aβ, in the context of the *Fmr1^KO^*, is necessary for many of the pathological phenotypes including AGS, anxiety, dendritic dysmorphogenesis and mGluR-LTD observed in the mice and that these effects are likely mediated by mGluR_5_ signaling. Furthermore, these data suggest a positive feedback loop whereby extracellular Aβ stimulates dendritic translation of AβPP through a mGluR_5_ signaling pathway providing more target for amyloidogenic processing and the generation of additional Aβ.

## Discussion

FXS is the most common form of inherited mental retardation and autism. It is caused by the loss of FMRP, an mRNA binding protein, which localizes to dendrites and regulates protein synthesis. Considerable effort has focused on characterizing the FMRP mRNA ligands and signaling pathways, particularly mGluR_5_ [36], that contribute to FXS phenotypes. We have demonstrated that *App* mRNA is an FMRP mRNA ligand whose translation is regulated through mGluR_5_ [11].

AβPP plays a critical physiological role in synapse formation and maintenance [37], [38] with expression increasing during neuronal differentiation, maximal during synaptogenesis and decline when mature connections are completed [39]–[42]. Maximal AβPP expression coincides with the critical period of sensory development in rodents (postnatal weeks 2–6) [43]. FMRP is also developmentally regulated in the neonatal brain where it peaks at the end of the first postnatal week and declines thereafter [44]. AβPP is processed by α-, β- and/or γ-secretases to produce soluble N-terminal domains of AβPP (sAβPPα and sAβPPβ), Aβ and C-terminal fragments. Aβ, which is over-expressed in AD and DS [45], is strongly implicated in impaired synaptic function and synapse loss observed early in the development of AD [46], [47], and we propose that their dysregulated production in FXS contributes to disease pathology. From conception, DS individuals over-express *APP* mRNA, AβPP and extracellular amyloid [48], [49]. Like FXS, DS patients show mental retardation, craniofacial abnormalities and dendritic dysmorphogenesis [1], [50]. Thus, increased AβPP and/or AβPP proteolytic products could provide a common effector at the molecular level for the neuroanatomic and behavioral phenotypes observed in all of these disorders [51].

We found that the peripheral concentration of Aβ_1–42_ and the Aβ_1–42_/Aβ_1–40_ ratio were significantly decreased in full-mutation FXS males compared to control donors. A reduced Aβ_1–42_/Aβ_1–40_ ratio is an independent risk factor for AD irrespective of the total Aβ load [52]–[58]. Plasma Aβ_1–42_ levels are increased in patients with mild cognitive impairment, but drop to control levels by the time of AD diagnosis [59]. In DS, elevated plasma Aβ_1–42_ is associated with earlier onset of AD [60] and the Aβ_1–42_/Aβ_1–40_ blood plasma ratio is lower than in controls [61]. Thus, our finding of a reduced Aβ_1–42_/Aβ_1–40_ ratio in FXS patients compared to control plasma is consistent with other amyloidogenic diseases.

The effectiveness of drug therapy in FXS is currently assessed exclusively by behavioral testing. Perhaps most importantly, the availability of a plasma biomarker for FXS may permit the monitoring of drug therapy as well as predict disease progression. Other studies have demonstrated reduced cAMP production in platelets [62] and delayed early-phase phosphorylation of ERK in lymphocytes [63] from FXS patients. Our data demonstrates that Aβ_1–42_ is significantly lower in FXS plasma than control subjects, and in conjunction with two studies demonstrating elevated sAβPPα in autistic children [16], [17], suggests that both AβPP and Aβ are viable biomarkers for FXS. We did not observe increased sAβPPα in adult FXS plasma suggesting that AβPP expression and processing decrease with age consistent with prior studies [16]. Previously reported control plasma levels of Aβ range from 130–208 pg/mL for Aβ_1–40_ and 15 pg/mL–85.7 ng/mL for Aβ_1–42_ [53], [58], [64], [65]. Thus, there is wide range of reported plasma Aβ_1–40_ and Aβ_1–42_ levels in the literature and our studies fall within that range. A critical question remains regarding if these catabolites are associated with disease severity or progression. There is a paucity of human FXS brain tissue available for analyses. Our preliminary studies indicate a trend for elevated Aβ in FXS brain, which agrees with data in *Fmr1^KO^* mice [11].

FXS patients exhibit hyper-reactivity to visual, olfactory, tactile and auditory stimulation [1], [66]–[69]. This hypersensitivity phenotype is manifested as AGS in *Fmr1^KO^* mice [20]. If Aβ contributes to AGS, then AD and DS mice would also be susceptible to seizures. Consistent with this, Tg2576, FRAXAD and DS mice, which all over-express hAβPP with the Swedish familial mutation and/or mouse AβPP, exhibit AGS [70]. *Fmr1^KO^/App^HET^* male mice exhibited a statistically significant decrease in AGS but not in WR whereas MPEP significantly reduced WR in *Fmr1^KO^* mice. These data suggest that the reduction of AβPP/Aβ in *Fmr1^KO^* mice is not particularly effective at reducing the induction of AGS, but does retard progression to clonic-tonic seizures. *Fmr1^KO^* mice also exhibit enhanced mGluR-LTD [34], which is lost in *Fmr1^KO^/App^HET^* mice. The *Fmr1^KO^* reduces mGluR-LTD in an *App^HET^* background, but enhances mGluR-LTD in an *App^WT^* background. The large difference in the maximal depression of synaptic transmission between *Fmr1^KO^*/*App^HET^* and *Fmr1^KO^* in opposite directions from WT mice, which exhibit equivalent mGluR-LTD as *App^HET^*, suggests that FMRP and AβPP/Aβ play important and synergistic roles in modulating mGluR-LTD. Thus, the over-expression of AβPP or an AβPP catabolite lowers seizure threshold and enhances mGluR-LTD, and approaches to attain normal synaptic levels of these proteins could prove therapeutic.

MPEP is a potent and highly selective noncompetitive antagonist of mGluR_5_ [71], [72] that reduces AGS, anxiety phenotypes and dendritic spine protrusion morphology in *Fmr1^KO^* [22], [26]. We have previously demonstrated that mGluR_5_ blockade inhibits translation of AβPP in synaptoneurosomes [11] and herein demonstrate reversion of several FXS phenotypes by genetic manipulation to reduce AβPP/Aβ. Minocycline, a second-generation tetracycline compound, reverts several FXS phenotypes [24], possibly by altered Aβ fibril formation [73] or Aβ-induced neuronal death and glial activation [74]. In aggregate, these data suggest that mGluR_5_ antagonists and minocycline therapies converge on a similar signaling pathway resulting in decreased Aβ levels/activity as obligatory for the rescue of FXS phenotypes.

How Aβ mediates synaptic dysfunction remains unclear. Aβ promotes AD-like cytoskeletal abnormalities and can promote intracellular accumulation of sAβPP in primary cultured neurons [75]. Soluble oligomers of Aβ increase LTD in WT hippocampal slices and inhibit long-term potentiation [76]–[80]. A similar enhancement of mGluR_5_-mediated LTD occurs in the hippocampus of *Fmr1^KO^* mice [34], and MPEP prevents the block in long-term potentiation [81]. Aβ causes membrane depolarization and calcium influx, activates mGluR_1_ [82] and functions as an extracellular scaffold for mGluR_5_ [83]. *In vitro*, Aβ_1–42_ altered the expression of important dendritic proteins regulated by FMRP including AβPP, Map1B and PSD95 with known roles in synaptogenesis and/or dendritic spine morphology [37], [38], [44], [84]. A previous report utilizing neuronal hybrid cells demonstrated that Aβ_1–40_ increases AβPP levels also supporting the existence of an Aβ-driven positive feedback loop [85]. Lower levels of PSD95 are observed in Tg2576 neurons, which constitutively overexpress Aβ and ADDLs [84]. In our hands, levels of the immediate-early gene Arc were not changed during a 1 hr stimulation with low molecular weight oligomers of Aβ_1–42_; however, others have observed that higher molecular weight Aβ oligomers (10–100 kDa) bind in a punctate pattern to the surface of neurons, colocalize with PSD95 and upregulate Arc [47]. We can block the Aβ-induced increase in AβPP levels with either MPEP or anisomycin suggesting that an mGluR_5_- and translation-dependent pathway is involved. The varied expression of known FMRP targets in response to Aβ suggests that dendritic translation can be modulated through FMRP-dependent and independent pathways. Aβ_1–42_ rapidly increases phosphorylated ERK levels suggesting that ERK mediates downstream signaling.

In conclusion, our work demonstrates that AβPP translation is regulated through an mGluR_5_/FMRP-mediated pathway. Excessive signaling through mGluR_5_ in the absence of FMRP leads to increased AβPP production and processing and we have observed elevated AβPP and Aβ levels in *Fmr1^KO^* mice [11]. Genetic reduction of AβPP levels in *Fmr1^KO^* mice has reverted or partially rescued FXS seizure, behavioral, dendritic spine and mGluR-LTD phenotypes. Furthermore, treatment of primary neurons with Aβ_1–42_ increased while anti-Aβ reduced dendritic AβPP expression suggesting that an Aβ-driven positive feedback loop drives synthesis/processing of AβPP through a mGluR_5_ signaling pathway. Our results have potential implications for the treatment of FXS as plasma AβPP/Aβ can be readily screened as biomarkers to evaluate potential therapies including mGluR_5_ antagonists as well as secretase inhibitors and anti-Aβ, which are currently undergoing testing for the treatment AD.

## Materials and Methods

### Ethics Statement

Adequate measures were taken to minimize pain or discomfort to the mice, and all husbandry, seizure and euthanasia procedures were performed in accordance with NIH and an approved University of Wisconsin-Madison animal care protocol administered through their Research Animal Resources Center (approval #G00468). Males with FXS and age-matched controls were recruited from the FXS Clinic at Rush University Medical Center (RUMC) in Chicago, IL. The study was approved by the RUMC Institutional Review Board and all donors or their legal guardians signed the appropriate consent forms for study participation.

### Mouse Husbandry

WT and *Fmr1^KO^* mice (C57BL/6 background) were bred and housed as previously described) [11]. *Fmr1^KO^* females [86] were crossed with *App^KO^* males [87] (Jackson Laboratories #004133, C57BL/6 background) to generate *Fmr1^HET^/App^HET^* females and *Fmr1^KO^/App^HET^* males that were crossed to generate *Fmr1^KO^/App^KO^* mice. For the behavioral testing, littermate controls were generated by crossing *Fmr1^KO^/App^HET^* females with *Fmr1^KO^/App^HET^* males to generate *Fmr1^KO^/App^WT^*, *Fmr1^KO^/App^HET^* and *Fmr1^KO^/App^KO^* progeny and by crossing *Fmr1^WT^/App^HET^* females with *Fmr1^WT^/App^HET^* males to generate *Fmr1^WT^/App^WT^*, *Fmr1^WT^/App^HET^* and *Fmr1^WT^/App^KO^* progeny. Genotypes were determined by PCR analysis of DNA extracted from tail biopsies. The WT, HET or KO state of the *App* gene was determined by genotyping with primer 1: 5′-CTG CTG CAG GTG GCT CTG CA-3′, primer 2: 5′-CAG CTC TAT ACA AGC AAA CAA G-3′, and primer 3: 5′-CCA TTG CTC AGC GGT GCT GTC CAT-3′ to generate a 250 base pair WT allele with primers 1 and 2 and a 470 base pair targeted KO allele with primers 2 and 3.

### Assessment of AβPP by Western Blot Analyses

Left hemispheres from WT, *Fmr1^KO^*, *App^HET^* and *Fmr1^KO^/App^HET^* mice (1 month old males; n = 3 per genotype) were homogenized in protein extraction buffer [10 mM Tris (pH 7.6), 2 mM EDTA, 150 mM NaCl, 1% Triton X-100, 0.25% NP-40, 1× protease inhibitor cocktail (Research Products International Corp., Mount Prospect, IL, USA, catalog #P50600)], mixed for 30 min at 4°C and spun at 12,000 rpm for 10 min at 4°C. The protein concentrations of the supernatants were determined by BCA assay (Thermo Fisher/Pierce, Rockford, IL, USA). Lysates (18.75 µg per lane) were separated by 12% SDS-PAGE, transferred to nitrocellulose and western blotted as previously described [11] with anti-AβPP antibody (Life Technologies Corporation, Carlsbad, CA, USA, catalog #51-2700; diluted 1∶250) and anti-tubulin (Santa Cruz Biotechnology, Santa Cruz, CA, catalog #sc-8035; diluted 1∶250). AβPP signals were normalized to tubulin and plotted as a percentage compared to WT levels. Error bars represent the SEM of three mice.

### Audiogenic Seizures

All mouse strains were tested at postnatal day 21, the peak of AGS sensitivity in C57BL/6. The experimental apparatus consisted of a clear, Plexiglas box (13″L×8″W×7″H) with the sound source located inside the box (LOUD KEY™ jogger's alarm). Mice were weighed and then placed individually into the center of the chamber and exposed to a siren that generated noise at 118 dB for 5 min. Loud, acoustic stimulation causes WR within 20–30 sec followed by erratic leaping, clonic convulsions and tonic hind limb extension by 40–50 sec followed by respiratory arrest and death [32]. The percentage of mice exhibiting WR, AGS and death were scored versus gender and genotype and assessed for statistical significance by the Fisher exact test. The *Fmr1^KO^/App^HET^* mice used in the AGS studies were offspring generated by crossing *Fmr1^KO^* females with *Fmr1^KO^/App^KO^* males to avoid effects due to maternal genotype in comparing *Fmr1^KO^* and *Fmr1^KO^/App^HET^*. MPEP was a kind gift from FRAXA Research Foundation (Newburyport, MA) and was dissolved at 1 mg/mL in DPBS before I.P. injection at 30 mg/kg body weight 30 min prior to AGS testing at age P21.

### Marble Burying

Mice were acclimated to the behavioral testing room for at least 15 min prior to transfer to a clean cage containing corn cob bedding and 20 black marbles arranged in a rectangular 4×5 grid over 2/3 of the cage. The mouse was placed into the cage at the end that did not contain marbles and allowed to explore the new cage with the marbles for 30 min after which the mouse was returned to its homecage and the number of visible marbles (more than half not buried) counted. Background white noise in the room was set to 70 dB. There is variability in this assay with some laboratories observing that *Fmr1^KO^* mice bury more marbles than WT (R. Paylor, personal communication); however, differences in behavioral outcomes can vary between facilities due to genetic background or altered environmental factors such as housing conditions and diet.

### Open Field

Mice were acclimated to the behavioral testing room for at least 30 min before placement into the center of a clear, Plexiglas chamber measuring 14.5″L×14.5″W×10″H. Mouse movement was monitored in the chamber for 15 minutes with LimeLight2 software interfaced with an overhead camera. The open field arena was arranged into a 16 square grid in the camera window with 4 squares in the center and 12 squares around the perimeter. Time spent in the center 4 squares (measure of anxiety) and total distance traveled (measure of locomotion) were compared between genotypes.

### DiI Labeling and Analyses of Dendritic Spines

Primary mouse neurons were prepared from embryonic (age E15–17) dissected brains from timed pregnant WT and *Fmr1^KO^* female mice as previously described) [11]. To generate *Fmr1^KO^/App^HET^* cells, *Fmr1^KO^* females were mated with *Fmr1^KO^/App^KO^* males. Cells were cultured for 15 days on poly(D)-lysine coated glass coverslips inside of 12-well tissue culture dishes, fixed with 4% paraformaldehyde and stained with lipophilic DiI dye (Life Technologies Corporation, Carlsbad, CA, USA). For the staining, the wells were aspirated and sprinkled with DiI crystals and a small amount of DBPS was added to the edge of the wells to prevent dehydration of the cells. Cells were stained for 10 min, copiously washed with DPBS to remove all crystals and fixed to slides with ProLong Gold Antifade (Life Technologies Corporation, Carlsbad, CA, USA). Slides were allowed to dry for at least 3 days to allow complete migration of the DiI into dendritic spines. Dendritic spines were imaged on a Zeiss Axioplan 2 Imaging Photomicroscope equipped with a MBF Biosciences automated XYZ stage and MicroFire A/R camera. Images were taken using the 100× objective (Zeiss FLUAR 100×/1.30 oil) and Zeiss Immersol™ 518F oil at ambient temperature. Spine length was quantitated with StereoInvestigator v9 software. Contours were drawn around the protrusions and the feret max (length) and feret min (widest width) of the contours were calculated. A minimum of 2–6 coverslips were analyzed per neuronal cell prep and images of neurons were taken from multiple areas of those coverslips. Data is representative of multiple batches of neuronal cells. A minimum of 746 spines were quantitated per genotype. The feret width was divided by feret max and protrusions having a ratio less than 0.5 were classified as filopodia and those with a ratio greater than or equal to 0.5 were classified as spines. For MPEP treatments, 2.5 µM MPEP was added to the neurons for the indicated times followed by washing the cells with DPBS and fixation and staining as previously described. A minimum of 263 protrusions were quantitated per MPEP treatment.

### Hippocampal Slice Preparation and mGluR-LTD Field Recordings

WT, *Fmr1^KO^*, *App^HET^* and *Fmr1^KO^/App^HET^* mice were rapidly decapitated by cervical dislocation (3-month old males). Hippocampal slices were prepared and electrophysiology performed as previously described [88]. After a 50 min stable baseline, slices were bathed in 50 µM S-DHPG for 10 min to induce mGluR-LTD. A 2 min trace of an average of four 30 sec traces was recorded over 100 min post-drug application and the slope of the fEPSP was measured and graphed as a function of time.

### Treatment, Staining and Immunofluorescence Analyses of Primary Neurons

For inhibitor treatments, cells were pretreated with vehicle, 2.5 µM MPEP or 40 µM anisomycin for 15 min prior to culture for 1 hr with vehicle or 20 nM Aβ_1–42_. Aβ_1–42_ (CalBiochem, catalog #171596) was prepared as previously described to generate oligomers, but not high molecular weight or fibrillar aggregates [89], [90]. For antibody treatments, 10 µg mouse IgG (Sigma #I5381) or anti-Aβ (Santa Cruz, catalog #28365LS) were added to 0.5 mL culture media inside transwells (Corning, catalog #3460, 0.4 µm pore size) that were situated above neuron-coated glass coverslips in 12-well tissue culture dishes. Each well contained 1 mL culture media and the transwell contained an additional 0.5 mL of culture media. Neurons were cultured for 3 days with the indicated antibodies prior to fixation and staining. Cells were stained overnight with: anti-22C11 against the amino-terminus of AβPP (Chemicon, catalog #MAB348, 1∶2000), anti-phosphERK (Santa Cruz, catalog #sc-23759, 1∶100), anti-Arc/Arg (Santa Cruz, catalog #sc-17839, 1∶100), anti-Map1B (Santa Cruz, catalog #58784, 1∶100), anti-RhoB (Santa Cruz, catalog #sc-180, 1∶100) and anti-PSD95 (Santa Cruz, catalog #sc-71935, 1∶100) followed by visualization with appropriate goat anti-mouse or anti-rabbit rhodamine-conjugated secondary antibodies (Invitrogen, 1∶500 for 30 min in the dark). Images were acquired with a Nikon C1 Laser Scanning Microscope (Nikon Eclipse E600 upright microscope) using the 543 Diode (1 mw Mellet Griot) laser, the Nikon Plan Apo 60×/1.40 oil objective with Zeiss Immersol™ 518F oil at ambient temperature, and Nikon EZ-C1, v3.91 software (Nikon Corp, Tokyo, Japan). Stained protein levels in the puncta of 4–7 dendrites per sample were quantitated with IMAGE J software using the Analyze Particles function (Rasband, W.S., Image J, U.S. National Institutes of Health, Bethesda, Maryland, USA, http://rsb.info.nih.gov/ij/, 1997–2006). Statistical significance was determined by one-way ANOVA and Student T-test analyses.

### Blood Collection

Males with FXS and age-matched controls were recruited from the FXS Clinic at Rush University Medical Center (RUMC) in Chicago, IL. All FXS subjects (ages 9–32 years old) were positive by DNA analyses for a fully methylated expansion mutation in the *FMR1* gene. Controls (age 23–33) were normal volunteers working at RUMC and had no history of cognitive or mental health disorders. The age and medications of the donors are listed in Table S1. Blood was drawn from donors into lithium heparin-coated blood collection tubes and spun at 1,500 rpm. The plasma supernatant was removed and frozen at −80°C. The anti-coagulated blood was mailed by overnight delivery from RUMC to the University of Wisconsin-Madison where PBMC were isolated within 24 hr.

### Assessment of AβPP/AβPPα, Aβ_1–40_ and Aβ_1–42_ by ELISA

Plasma was thawed and clarified at 12,000 rpm for 10 min at 4°C prior to ELISA assays for AβPP/AβPPα, Aβ_1–40_ and Aβ_1–42_ per the manufacturer's instructions (BioSource #KHB0051, KHB3482, KHB3442) with the following modifications for the Aβ assays: (1) the sample volume was doubled from 50 µL to 100 µL, (2) the incubation time was extended from 3 hr to overnight at 4°C, and (3) after the overnight incubation, the samples were removed from the antibody-coated wells prior to addition of the detection antibody. PBMC were isolated as previously described [91] and cultured for 24 hr prior to harvesting the cells and culture media for ELISA analyses. Hippocampus and neocortex (pre and post central gyri) samples were sectioned from left cerebral hemispheres of control and FXS brain autopsy tissue. Detergent-soluble lysates were prepared as previously described) [11] for analyses of AβPP and Aβ_1–40_.

### Statistical Analyses

One-way ANOVA was performed using GraphPad Prism version 5.0 d for Mac OS X (GraphPad Software, San Diego, CA) to compare the means of three or more unmatched groups for the behavioral and dendritic spine analyses. Student T-test analyses were used to quantitate statistical significance for the western blotting, marble burying, open field, dendritic spine protrusion length, immunofluoresence and ELISA data. Fisher exact tests were used to quantitate statistical significance for the AGS data. Two-way ANOVA with Bonferroni posthoc tests was used to quantitate statistical significance for the mGluR-LTD analyses.

## Supporting Information

Figure S1 **Assessment of hippocampal slice health.** Hippocampal slices from WT (black circles), *Fmr1^KO^* (white circles), *App^HET^* (black triangles) and *Fmr1^KO^/App^HET^* (white triangles) mice exhibit equivalent synaptic transmission as determined by the input/output relationship (A) and ppf (B). i/o was also measured at the end of the recordings to assess slice health and showed similar i/o relationships to those shown in (A) (data not shown). (B) fEPSP size as percent of first stimulus versus interstimulus interval (msec). Data were analyzed by two-way ANOVA/Bonferroni multiple comparison analyses. There were no statistically significant differences in the i/o relationships. The WT ppfs were statistically different (p<0.05) from *App^HET^* (many time points), WT versus *Fmr1^KO^* (50 msec time point); WT versus *Fmr1^KO^/App^HET^* (first four time points). There were no other statistically significant differences in ppfs for the remaining datasets.
(TIFF)

Figure S2 **MPEP and Aβ_1–42_ alter dendritic phosphoERK levels.** (A) WT and *Fmr1^KO^* neurons were treated with 10 µM MPEP [one-way ANOVA p<0.05, F = 2.1], and (B) WT neurons were treated with 20 nM Aβ_1–42_ for the indicated times prior to fixation and staining with anti-phosphoERK [one-way ANOVA p<0.0001, F = 13]. Stars (★) denote statistically different results by Student T-test analyses (p<0.05).
(TIFF)

Table S1 **Age and medications of donors.**
(XLS)
