# Supplementary figures and images for: Reversal of Fragile X Phenotypes by Manipulation of AβPP/Aβ Levels in Fmr1KO Mice
Source: PLoS One. 2011 Oct 26;6(10):e26549. doi: 10.1371/journal.pone.0026549 (PMC3202540; doi:10.1371/journal.pone.0026549)

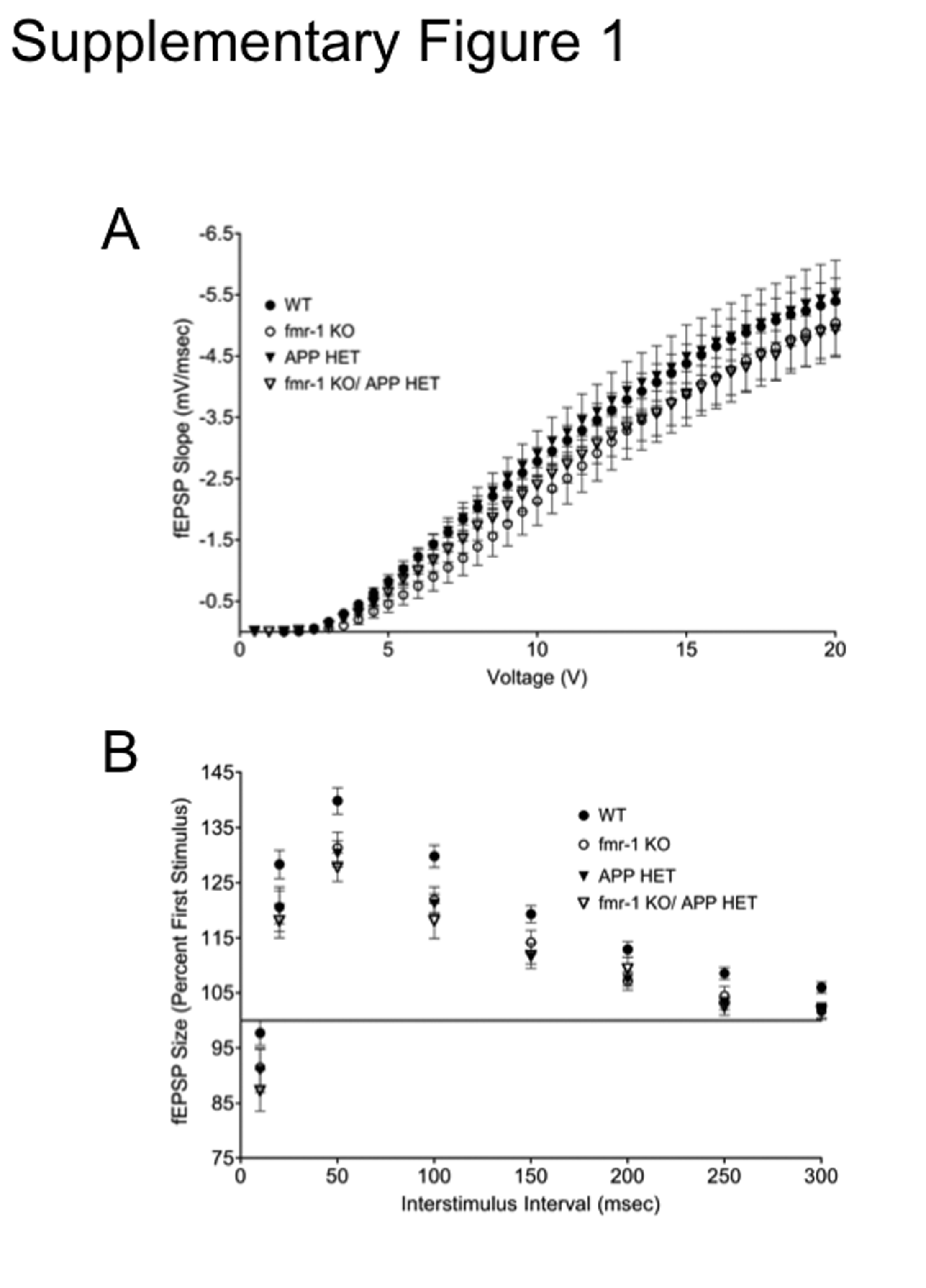

Supplement: Figure S1 — Assessment of hippocampal slice health. Hippocampal slices from WT (black circles), Fmr1KO (white circles), AppHET (black triangles) and Fmr1KO/AppHET (white triangles) mice exhibit equivalent synaptic transmission as determined by the input/output relationship (A) and ppf (B). i/o was also measured at the end of the recordings to assess slice health and showed similar i/o relationships to those shown in (A) (data not shown). (B) fEPSP size as percent of first stimulus versus interstimulus interval (msec). Data were analyzed by two-way ANOVA/Bonferroni multiple comparison analyses. There were no statistically significant differences in the i/o relationships. The WT ppfs were statistically different (p<0.05) from AppHET (many time points), WT versus Fmr1KO (50 msec time point); WT versus Fmr1KO/AppHET (first four time points). There were no other statistically significant differences in ppfs for the remaining datasets. (TIFF) [file pone.0026549.s001.tiff]

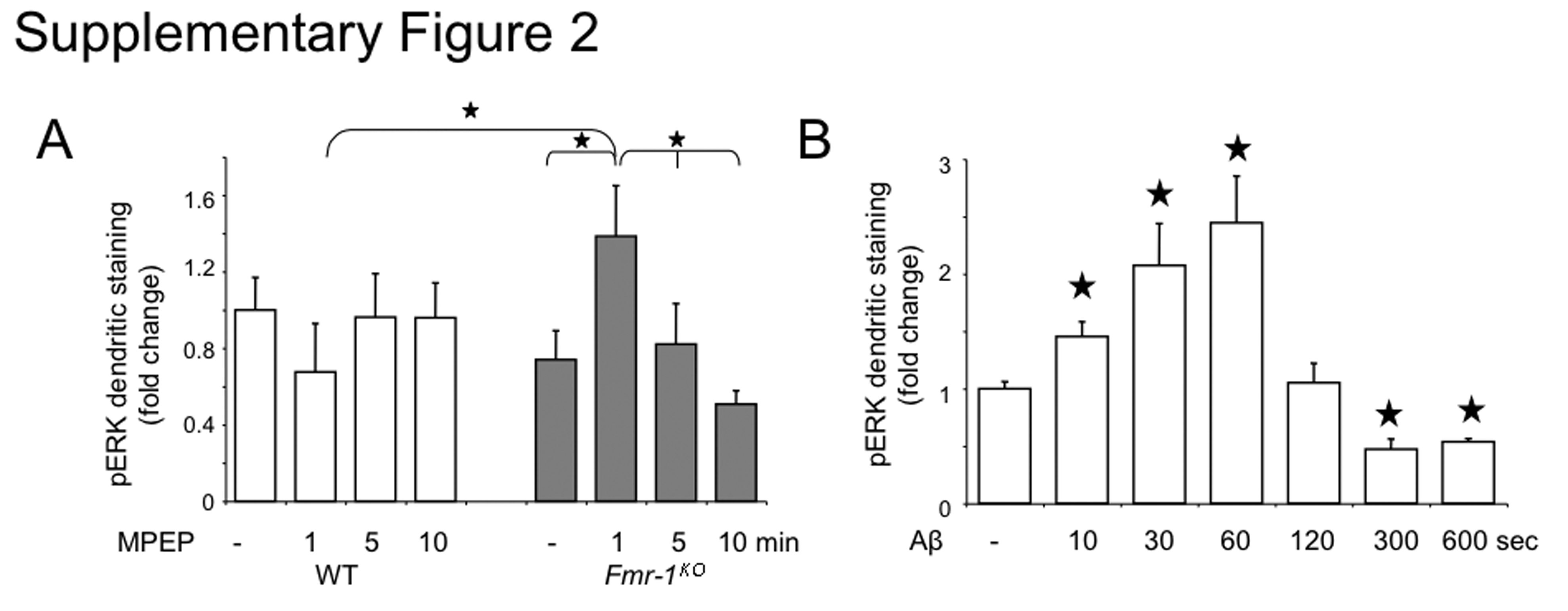

Supplement: Figure S2 — MPEP and Aβ1–42 alter dendritic phosphoERK levels. (A) WT and Fmr1KO neurons were treated with 10 µM MPEP [one-way ANOVA p<0.05, F = 2.1], and (B) WT neurons were treated with 20 nM Aβ1–42 for the indicated times prior to fixation and staining with anti-phosphoERK [one-way ANOVA p<0.0001, F = 13]. Stars (★) denote statistically different results by Student T-test analyses (p<0.05). (TIFF) [file pone.0026549.s002.tiff]
